# Stepping into Perpetrators’ Shoes: How Ingroup Transgressions and Victimization Shape Support for Retributive Justice through Perspective-Taking With Perpetrators

**DOI:** 10.1177/0146167219858652

**Published:** 2019-06-27

**Authors:** Mengyao Li, Bernhard Leidner, Silvia Fernandez-Campos

**Affiliations:** 1 University of Massachusetts Amherst, Amherst, MA, USA; 2 Max Planck Institute for Research on Collective Goods, Bonn, Germany; 3 New School for Social Research, New York, NY, USA

**Keywords:** perspective-taking, moral disengagement, intergroup conflict, justice, ingroup identification

## Abstract

Three experiments (total *N* = 1,061) examined the morally disengaging function of perspective-taking with ingroup perpetrators in intergroup conflict. In the context of the Iran–U.S. conflict, Americans who strongly identified with their country showed increased perspective-taking with perpetrators, which in turn led to reduced support for retributive justice in response to the perpetration rather than suffering of intergroup violence (Experiment 1; *N* = 191). Experiment 2 (*N* = 294) replicated these findings in the context of the Israel–Syria conflict with Israeli Jews and demonstrated that perspective-taking with ingroup perpetrators serves a similar function as moral disengagement. Experiment 3 (*N* = 576) manipulated perpetrator perspective-taking, demonstrating its causal effect on support for retributive justice, again moderated by ingroup identification. The negative implications of understanding perpetrators for addressing intergroup transgressions are discussed.

The ability to imagine how others think and feel is arguably one of the most intriguing and sophisticated attributes of human nature. For various reasons and to various degrees, people try to contemplate another person’s point of view, which is largely beneficial for social relations. Adopting another’s perspective can foster a sense of psychological connectedness between the self and the perspective-taking target (Davis, Conklin, Smith, & Luce, 1996; Galinsky & Moskowitz, 2000) and promote prosocial and altruistic behavior (Toi & Batson, 1982). Very little of this work, however, has examined a less intuitive, yet not uncommon, case of perspective-taking: the attempt to understand moral transgressions and those who commit them. In the current contribution, we provide empirical evidence for the argument that group members can be motivated to take the perspective of ingroup perpetrators, and such perspective-taking has the potential to hinder efforts to restore justice, particularly in the form of retributive justice.

According to Baumeister’s (1997, 2012) analysis of evil, attempts to understand the perpetrators’ perspective risk seeing their crimes as less heinous and the perpetrators as less responsible for the crimes. Perpetrators tend to minimize the harm they have committed, and thus any effort to understand the situation from their perspective might fall into the trap of following similar “minimalist, distancing styles of thought” (Baumeister, 1997, p. 4). This potential pitfall of people’s attempts to take perpetrators’ perspective is especially worth discussing in the context of intergroup transgressions. When the interests of one’s own group are at stake—for instance, when ingroup members have committed violence against outgroup members—individuals may even be motivated to appraise the transgression from the perpetrators’ perspective in an effort to make sense of or even justify it, thereby protecting the ingroup’s moral image. When the ingroup has been victimized by an outgroup, on the contrary, such perpetrator perspective-taking seems rather unlikely. Despite the importance of understanding perspective-taking in intergroup contexts, the majority of prior research has focused on interpersonal perspective-taking and, in particular, perspective-taking with victimized, marginalized, and negatively stereotyped groups (e.g., Batson et al., 1997; Dovidio et al., 2004; Galinsky & Moskowitz, 2000; Vescio, Sechrist, & Paolucci, 2003). The current research thus aimed to extend the existing literature to intergroup domains and to explore the role of perspective-taking with perpetrators in addressing intergroup violence. We hypothesized that when the perpetrators belong to one’s own group, attempts to understand their perspective are driven by ingroup-defensive motives and may serve a morally disengaging function, leading to reduced support for punitive justice efforts.

## Perspective-Taking With Perpetrators

While the consequences of victim perspective-taking are reasonably well-understood, much less is known about the implications of perspective-taking with perpetrators of moral transgressions. In the context of interpersonal interactions, victims and bystanders who adopt the perspective of transgressors, compared to those who do not, tend to make more situational rather than dispositional attributions, experience more benevolent emotions, and are more likely to accept apologies from the transgressor (Experiment 3 in Batson et al., 1997; Takaku, 2001; Takaku, Weiner, & Ohbuchi, 2001). Extending these findings to the intergroup context, Exline, Baumeister, Zell, Kraft, and Witvliet (2008) found that seeing one’s own group’s capability for wrongdoing predicted forgiveness of outgroup transgressors through reduced perceived severity of the offense, increased empathic understanding of and perceived similarity to the transgressors. While these findings provide evidence that victims’ efforts to appreciate the perspective of perpetrators can foster positive intergroup attitudes, Lucas, Galinksy, and Murnighan (2016) offered a more nuanced account of perspective-taking and its effects on attitudes toward moral transgressions. Perspective-taking with perpetrators, as their findings show, can either decrease or increase moral condemnation depending on the nature of the intentions attributed to the perpetrator.

Despite the generally positive consequences of perpetrator perspective-taking among victims, people’s natural and immediate response to ingroup victimization is perhaps not to understand their perpetrators’ feelings and motives. When the ingroup is responsible for the moral violation, on the contrary, the need to defend the ingroup might motivate group members to make sense of the perpetrators’ perspective, which could pave the way to justifying the immoral acts and possibly even exonerating the ingroup perpetrators from them. This tendency in perspective-taking with perpetrators, we argue, might be a key mechanism underlying victim and perpetrator group members’ divergent attitudes toward justice in the aftermath of intergroup violence.

## Perpetrator Perspective-Taking and Retributive Justice

When faced with large-scale violence and injustices, people tend to adopt vastly different responses depending on the specific role that their group played in the conflict (e.g., Leidner, Castano, Zaiser, & Giner-Sorolla, 2010; Li, Leidner, Petrović, Orazani, & Rad, 2018; Shnabel, Nadler, Ullrich, Dovidio, & Carmi, 2009). Although members of victim groups have a strong desire for justice and retribution (Leidner, Castano, & Ginges, 2013; Li et al., 2018; Lickel, Miller, Stenstrom, Denson, & Schmader, 2006), members of perpetrator groups are often motivated to morally disengage from ingroup-committed transgressions and resist efforts to achieve justice (Castano & Giner-Sorolla, 2006; Leidner et al., 2010; Roccas, Klar, & Liviatan, 2006). While past research has focused primarily on the divergent psychological needs of victim and perpetrator groups (e.g., Shnabel et al., 2009), less attention has been devoted to the more basic cognitive underpinnings of their differential responses to intergroup conflict. The notion that members of the perpetrator group actively engage in various moral disengagement strategies (Bandura, 1999) implies a basic, motivated cognitive process, in which individuals appraise the intergroup situation from the perspective of their fellow group members who committed immoral acts. We argue that when driven by ingroup-defensive motivations, adopting the perspective of ingroup perpetrators can facilitate justification of the ingroup’s immoral acts and thus serves a similar function as moral disengagement. Such motivated perspective-taking should therefore predict reduced support for justice efforts.

It is important to note that in this research we focused on the retributive aspect of justice. In the justice literature, a meaningful distinction has been made between retributive and restorative justice (e.g., Okimoto, Wenzel, & Feather, 2011). Although retributive justice is primarily concerned with unilateral punishment of perpetrators, restorative justice emphasizes bilateral efforts to restore the relationship between adversarial parties, for instance through reaffirming their shared values. Due to the different foci of retributive and restorative justice, we argue that perspective-taking with perpetrators contributes to the asymmetric reactions between perpetrator and victim group members, particularly regarding their support for retributive justice.

## The Moderating Role of Ingroup Identification

**Figure 1.**
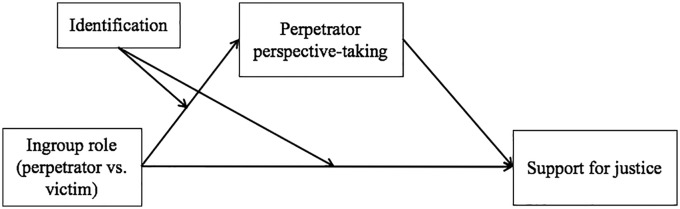
Conceptual model depicting the effects of ingroup role (perpetrator vs. victim) on support for justice through perspective-taking with perpetrators, moderated by ingroup identification.

Not everyone, however, responds defensively to ingroup-committed transgressions, nor does everyone respond vengefully to ingroup-suffered transgressions. Substantial research on social identification has demonstrated that people are more motivated to hold a positive view of their group to the extent that they identify with that group (e.g., Doosje, Ellemers, & Spears, 1995). As a consequence, when confronted with negative aspects of the ingroup, such as ingroup-committed transgressions, individuals who strongly identify with their group are less likely to accept the negative portrayal of their group (Branscombe, Ellemers, Spears, & Doosje, 1999). Due to high identifiers’ strong need to defend the ingroup, they should be particularly motivated to adopt the perspective of perpetrators when their ingroup has committed (rather than suffered) violence. Low identifiers, on the contrary, should be unlikely to exhibit the same bias in perspective-taking based on their ingroup’s role in the conflict. Moreover, the extent to which people take the perspective of perpetrators should in turn predict differential support for retributive justice among members of victim and perpetrator groups (see Figure 1 for the full conceptual model).

Recent research on social identification proposes a multidimensional view of group identification, for instance distinguishing between ingroup attachment and glorification (Roccas et al., 2006). Whereas attachment refers to one’s commitment to the ingroup and perceived importance of the group membership to one’s self concept, glorification refers to beliefs in the ingroup’s superiority over outgroups and unconditional loyalty to ingroup norms and authorities. These two dimensions of identification have different implications for intergroup attitudes and behavior. Compared to attachment, glorification has been shown to be a stronger motivator of ingroup-defensive reactions such as the use of exonerating cognitions, victim dehumanization, and reluctance to pursue justice when the ingroup is responsible for intergroup violence (e.g., Leidner et al., 2010; Roccas et al., 2006). Following this literature, the current research also adopts the bidimensional conceptualization of identification. Unlike exonerating cognition and victim dehumanization, however, taking the perpetrators’ perspective is not an explicit form of moral disengagement. It seems plausible that strong attachment to the ingroup might be sufficient to motivate perspective-taking with ingroup, rather than outgroup, perpetrators. Thus, we tested whether ingroup identification in general (glorification and attachment combined) would moderate the effects of the ingroup’s role in the conflict (perpetrator or victim) on perpetrator perspective-taking.

## Overview

We conducted three experiments to examine the morally disengaging function of perpetrator perspective-taking in two different international contexts: the tensions between the U.S. and Iran, and between Israel and Syria. Studies 1 and 2 tested the mediating role of perpetrator perspective-taking in the effects of ingroup perpetration versus victimization on group members’ support for retributive justice and the moderating role of ingroup identification. Study 3 experimentally induced perspective-taking with perpetrators, thereby providing evidence for its causal effects on support for retributive justice. Together, the present work makes a first attempt to integrate two previously disconnected lines of research: perspective-taking and moral disengagement. Furthermore, it contributes to the intergroup literature by examining perpetrator perspective-taking and its implications for retributive justice from both victim and perpetrator perspectives.

## Study 1

Study 1 tested the hypothesis that high (but not low) ingroup identifiers would support less retributive justice in response to ingroup perpetration than victimization and that high identifiers’ perspective-taking with ingroup perpetrators would explain why such a justice bias occurs. In the context of the conflict between the United States and Iran, we experimentally manipulated whether American citizens have committed or suffered human rights violations. The manipulation enabled us to investigate the extent to which Americans reacted differently to intergroup transgressions depending on their ingroup’s role in the conflict.

### Method

#### Participants

The sample consisted of 191 American adults recruited online through Amazon’s Mechanical Turk (MTurk).^1^ To determine sample size, we followed a general rule of approximately 100 participants per cell, which guarantees high power (>.80) to detect two-way interactions with small to medium effect sizes. Our screening of the data quality indicated that 19 participants did not pay sufficient attention to the manipulation material, as indicated by their incorrect written summaries of the material, false answers to the manipulation check questions, and the little time they spent on reading the material. Two participants spent significantly more time on the reading material than the rest of the sample, suggesting that they may have been distracted during the reading task. Three participants raised suspicions about the credibility of the material. Four participants reported to have close Iranian family members or friends. The data from the remaining 163 participants were used in the subsequent analyses (69% women; age *M* = 34, *SD* = 11.48).

#### Procedure

Participants were randomly assigned to read a fictitious, but allegedly real, 2012 New York Times article depicting cases of prisoner abuse in an underground prison at the Afghan–Iranian border. In the ingroup transgression condition, participants read about American soldiers capturing and torturing Iranian civilians, whereas in the ingroup victimization condition participants read about Iranian soldiers capturing and torturing American civilians. The reported acts of abuse included sleep deprivation, severe beatings, suffocation, and humiliating acts. In one of the three cases, mistreatment and torture eventually led to the prisoner’s death. The news articles were identical across conditions except for the nationalities of the perpetrators and the victims. After the reading task, participants indicated the nationalities of the perpetrators and victims described in the article as a manipulation check. To ensure that they read the article carefully, they also summarized it in their own words. Then, participants completed the following measures on 9-point scales (1 = *strongly disagree*; 9 = *strongly agree*), in the order outlined below. Finally, participants completed routine demographic questions and were fully debriefed.

#### Materials

##### Perspective-taking with perpetrators

Adapted from the perspective-taking subscale of the Interpersonal Reactivity Index (Davis, 1980), five items measured the extent to which American participants took the perspective of the perpetrators in the news article (e.g., “*I tried to understand these American/Iranian soldiers better by imagining how things looked from their perspective*.”).

##### Support for retributive justice

Adapted from Leidner et al. (2013), five items measured the retributive aspects of justice, for example, “*To restore justice, the U.S./Iran need(s) to be punished for its actions described in the news report.*”

##### Ingroup identification

Ingroup identification was measured using the ingroup attachment and glorification subscales (Roccas et al., 2006). The *ingroup attachment* subscale contained eight statements, tapping the importance of the United States to participants’ identity and their commitment to the United States (e.g., “*Being American is an important part of my identity.*”). The *ingroup glorification* subscale contained eight statements, tapping participants’ belief in the superiority of the United States and their deference to American authorities (e.g., “*The U.S. is better than other nations in all respects.*”). Following others (e.g., Leidner & Castano, 2012; Leidner et al., 2010), the identification subscales were administered at the end of the study in order to avoid raising participants’ suspicion about the study goal. The attachment and glorification items were combined to form a composite score for ingroup identification.

### Results

#### Ingroup identification

Consistent with previous research, the manipulation did not have a significant effect on identification (α = .95, *M* = 5.58, *SD* = 1.73), *F*(1, 161) = 0.16, *p* = .686, η^2^*p* < .001.^2,3^ We were thus able to use ingroup identification, together with condition, as independent variables (IVs) in subsequent general linear models (GLMs) carried out in SAS 9.4. To this end, identification was standardized (Aiken & West, 1991). We also conducted the same analyses with glorification as a moderator while controlling for attachment and with attachment as a moderator while controlling for glorification. Both analyses produced similar patterns of results as reported below, suggesting that glorification and attachment played the same moderating role in the effects of condition on the dependent variables (DVs).

#### Joint effects of ingroup role and identification

##### Perspective-taking with perpetrators

**Figure 2.**
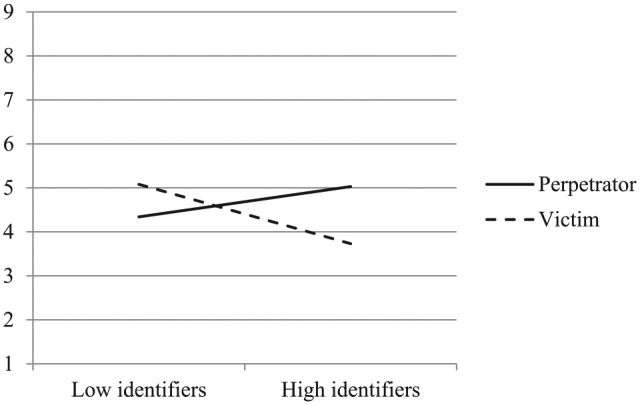
Perspective-taking with perpetrators as a function of ingroup’s role as perpetrator or victim and national identification (Study 1).

Analysis with perspective-taking with perpetrators (α = .92, *M* = 4.57, *SD* = 2.11) as the DV yielded a significant two-way interaction between ingroup role and identification (see Figure 2), *F*(1, 159) = 9.44, *p* = .003, η^2^*_p_* = .06, 90% confidence interval (CI) [.01, .12]. Simple effects revealed that high identifiers (1 *SD* above the mean) were significantly more likely to take the perspective of perpetrators when their ingroup was the perpetrator (*M* = 5.02) rather than the victim (*M* = 3.73), *t*(162) = 2.85, *p* = .005. Low identifiers (1 *SD* below the mean), in contrast, did not differ significantly in perpetrator perspective-taking, *t*(159) = −1.51, *p* = .133. If anything, they tended to be less likely to take the perspective of perpetrators when their ingroup was the perpetrator (*M* = 4.38) rather than the victim (*M* = 5.07). The main effects of ingroup role and identification did not reach significance, *Fs*(1, 159) < 1.20, *ps* > .270, η^2^*_p_s* < .01.

##### Support for retributive justice

**Figure 3.**
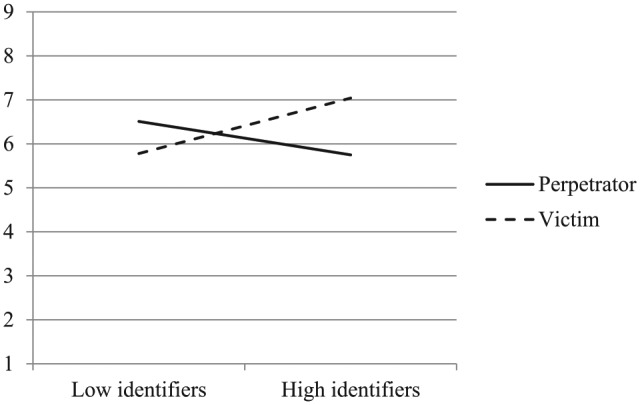
Support for retributive justice as a function of ingroup’s role as perpetrator or victim and national identification (Study 1).

As predicted, analysis with support for retributive justice (α = .88, *M* = 6.22, *SD* = 1.71) as the DV yielded a significant two-way interaction between ingroup role and identification (see Figure 3), *F*(1, 159) = 15.58, *p* < .001, η^2^*_p_* = .09, 90% CI [.03, .16]. Simple effects revealed that high identifiers supported significantly less retributive justice when the ingroup was the perpetrator (*M* = 5.74) rather than the victim (*M* = 6.98), *t*(159) = −3.40, *p* = .001. On the contrary, low identifiers were more supportive of retributive justice when the ingroup was the perpetrator (*M* = 6.52) rather than the victim (*M* = 5.72), *t*(159) = 2.19, *p* = .030. Again, no main effects reached significance, *Fs*(1, 159) < 1.00, *ps* > .340, η^2^*_p_s* < .01.

#### The mediating role of perpetrator perspective-taking

**Figure 4.**
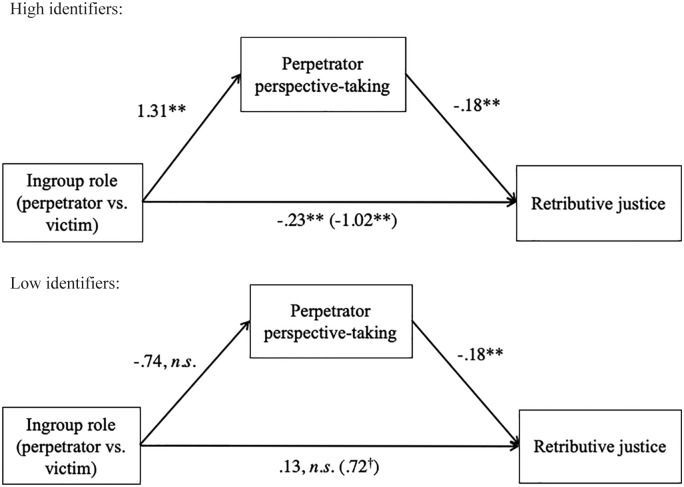
Moderated mediation models for high and low identifiers (Study 1). *Note.* All coefficients are unstandardized.

To test whether perspective-taking with perpetrators mediated the effect of ingroup role by identification on support for retributive justice, we conducted a moderated mediation analysis, in which ingroup role was introduced as the IV, perspective-taking as the mediator, identification as the moderator, and retributive justice as the DV (Hayes, 2018, model 8; see Figure 4 for all path coefficients at high and low levels of identification). In line with our mediational hypothesis, there were significant indirect effects of ingroup role on justice support through perspective-taking with perpetrators at high levels of identification (+1 *SD*), *boot coefficient* = −.23, 95% CI [–.530, –.029]. The same indirect effect was not significant at low levels of identification (−1 *SD*); if anything, it was trending in the opposite direction, *boot coefficient* = .13, 89% CI [.001, .314]. Importantly, the index of moderated mediation was significant, *boot coefficient* = −.18, 95% CI [–.400, –.027].

### Discussion

Study 1 demonstrated that individuals who strongly identified with their own group showed increased perspective-taking with perpetrators and reduced support for retributive justice in response to violence committed rather than suffered by the ingroup. By contrast, low identifiers exhibited the opposite pattern, taking somewhat less perspective of perpetrators and demanding more justice when their ingroup has committed rather than suffered violence. Although our predictions did not focus on low identifiers, these findings were consistent with past research conducted in similar intergroup contexts where low glorifying Americans tend to exhibit rather conciliatory and even ingroup-critical attitudes toward moral transgressions committed by the U.S. military (e.g., Leidner & Castano, 2012).

It is worth noting that the perspective-taking measure was anchored to the direct perpetrators of prisoner abuse, whereas the retributive justice measure was anchored to the perpetrator group (the United States or Iran). We made this methodological choice because while we were particularly interested in group-level punishment, perspective-taking is more meaningful when applied to “people” rather than “countries.” Although these two entities are often viewed differently, they arguably overlap to a considerable extent when ingroup-outgroup distinctions are made salient in a conflict situation, especially when the “people” in the current context are part of the official military that represents the country.

The analyses of indirect effects further indicated that perspective-taking with perpetrators played a role in explaining the diametrically opposed effects of ingroup victimization versus transgression on support for group-level retributive justice among both high and low identifiers. These results thus provided initial evidence for our conceptualization of perspective-taking as a motivated cognition—that is, high identifiers are motivated to adopt the perspective of ingroup perpetrators in an effort to exonerate the ingroup from punishment. Although reduced support for retributive justice can be attributed to moral disengagement (Leidner et al., 2010), we did not measure moral disengagement directly. It thus remained unclear whether perpetrator perspective-taking indeed served a similar function as moral disengagement. Study 2 addressed this issue in a different conflict context.

## Study 2

The main goals of Study 2 were threefold. First, we aimed to conceptually replicate the main findings of Study 1 in a different intergroup context: the conflict between Israel and Syria. Against the backdrop of the 1973 Yom Kippur War, we examined Israelis’ reactions to moral transgressions either perpetrated by Israeli soldiers against Syrian civilians, or by Syrian soldiers against Israeli civilians. Second, Study 2 included a measure of moral disengagement to provide more direct evidence for the morally disengaging function of perpetrator perspective-taking. In this study, we focused on one specific form of moral disengagement: the use of exonerating cognitions in response to moral transgressions (Roccas et al., 2006). If perpetrator perspective-taking indeed functions as a moral disengagement mechanism, it should correlate highly and positively with the use of exonerating cognitions. By the same token, both should predict reduced support for justice in reaction to ingroup-committed (rather than suffered) atrocities. While we did not formulate a strong hypothesis about perspective-taking and exonerating cognitions occurring one after the other in sequence, we tested our aforementioned predictions through serial multiple mediator models (Hayes, 2018). This statistical approach takes into account the interrelation and functional similarity of perspective-taking and exonerating cognitions. Finally, Study 2 also tested whether taking the perspective of perpetrators contributed to perpetrator and victim group members’ differential support for retributive, but not restorative, justice.

### Method

#### Participants

A total of 294 Jewish Israelis currently living in Israel were recruited via the Midgam Project (www.midgam.com). Following the same data screening procedure in Study 1, 48 participants were excluded. The data from the remaining 246 participants were used in the subsequent analyses (50% women; age *M* = 40.89, *SD* = 14.80).

#### Procedure

After consenting to participate in a study on “Conflicts in the Middle East,” participants were randomly assigned to read one of two fictitious, but allegedly real, Ynet news articles. Similar to the manipulation materials used in Study 1, the articles described cases of prisoner abuse in a secret facility that was established by either the Israeli or Syrian military near the Syrian–Israeli border during the Yom Kippur War. In the ingroup transgression condition, participants read about Israeli soldiers capturing and torturing Syrian civilians, whereas in the ingroup victimization condition participants read about Syrian soldiers capturing and torturing Israeli civilians. After the reading task, participants completed attention and manipulation checks, and the following measures on 9-point scales (1 = *strongly disagree*; 9 = *strongly agree*).

#### Materials

Perspective-taking with perpetrators was measured with the same five items as in Study 1, adapted to the Israeli–Syrian context (α = .90, *M* = 4.11, *SD* = 2.50).

##### Exonerating cognitions

Adapted from Roccas et al. (2006), five items (α = .84, *M* = 4.28, *SD* = 2.17) measured the use of exonerating cognitions in response to the events described in the article (e.g., “*The language that the news article used to describe the actions of the Israeli/Syrian military is too harsh.*” “*The Syrian/Israeli detainees deserved the treatment they received.*”).

##### Support for justice

*Retributive justice* was measured using the same items as in Study 1, adapted to the Israeli–Syrian context (α = .98, *M* = 4.73, *SD* = 3.02). Taken from Leidner et al. (2013), *restorative justice* was measured with five items (α = .81, *M* = 5.97, *SD* = 2.16) tapping apologetic behavior, financial reparation, and reaffirmation of shared values as ways to restore justice (e.g., “*Without a sincere apology from [Syria/Israel] for having acted wrongly, the injustice is not completely restored*”; “*To restore justice, the [Israeli/Syrian] detainees and their family members needs to receive financial compensation from [Syria/Israel] for what happened in the prison*”; “*To restore justice, Israel and Syria need to agree on rules of a peaceful world.*”).

Ingroup identification was measured with the same attachment and glorification subscales as in Study 1, adapted to the Israeli context (α *=* .93, *M =* 6.92, *SD =* 1.50).

### Results

#### Joint effects of ingroup role and identification.^4^

##### Perspective-taking with perpetrators

Analysis with perspective-taking with perpetrators as the DV yielded a significant main effect of condition, *F*(1, 241) = 211.50, *p* < .001, η^2^*p* = .47, 90% CI [.39, .53]. Participants were more likely to take the perpetrator’s perspective in the ingroup-perpetrator (*M* = 5.87) than the ingroup-victim condition (*M* = 2.51). As predicted, the main effect of condition was further qualified by a significant interaction with identification (see Figure 1 in the Supplementary Document), *F*(1, 241) = 9.54, *p* = .002, η^2^*p* = .04, 90% CI [.01, .08]. Analyses of simple effects revealed that high identifiers were significantly more likely to take the perspective of perpetrators when their ingroup was the perpetrator (*M* = 6.56) rather than the victim (*M* = 2.45), *t*(241) = 12.31, *p* < .001. Low identifiers, in contrast, exhibited the same pattern but to a much lesser degree (*M_perpetrator_* = 5.21, *M_victim_* = 2.57), *t*(241) = 8.01, *p* < .001. The main effect of identification was also significant, *F*(1, 241) = 6.85, *p* = .009, η^2^*p* = .03, 90% CI [.004, .07], indicating that identification was positively correlated with perpetrator perspective-taking, β = .13.

##### Exonerating cognitions

**Figure 5.**
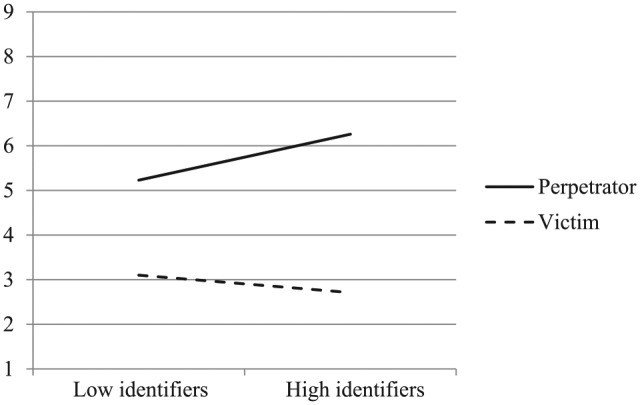
The use of exonerating cognitions as a function of ingroup’s role as perpetrator or victim and national identification (Study 2).

Analysis using exonerating cognitions as the DV also yielded a significant main effect of condition, *F*(1, 238) = 191.72, *p* < .001, η^2^*p* = .45, 90% CI [.37, .51]. Participants were more likely to use exonerating cognitions in response to ingroup perpetration (*M* = 5.74) than victimization (*M* = 2.90). Again, the main effect of condition was further qualified by an interaction with identification (see Figure 5), *F*(1, 238) = 11.54, *p* = .001, η^2^*p* = .05, 90% CI [.01, .10]. Simple effects analyses revealed that high identifiers were significantly more likely to use exonerating cognitions when their ingroup was the perpetrator (*M* = 6.26) rather than the victim (*M* = 2.71), *t*(238) = 12.03, *p* < .001. Low identifiers, in contrast, exhibited the same pattern but to a much lesser degree (*M_perpetrator_* = 5.23, *M_victim_* = 3.10), *t*(238) = 7.34, *p* < .001. The main effect of identification was not significant, *F*(1, 238) = 2.37, *p* = .125, η^2^*p* = .01, 90% CI [.000, .04].

##### Support for justice

Participants’ support for retributive justice was strongly correlated with their support for restorative justice, *r* = .74, *p* < .001. We thus conducted an exploratory factor analysis (EFA) to examine whether these two types of justice indeed constituted two distinct constructs in our data. Parallel analysis and screen plot were employed to determine the appropriate number of factors to retain. Both the parallel analysis and the screen plot indicated a two-factor solution. After applying an oblique rotation, all five retributive justice items and three restorative justice items tapping support for financial reparations and apologies as means to restore justice loaded onto one factor, while the other two restorative justice items tapping reaffirmation of shared values defined the second factor. This factor solution suggested that participants construed financial reparations and apologies as punitive, similar to retributive justice. Following the EFA, we created a new retributive justice variable, encompassing both the original five retributive items and the three restorative items (α = .97, *M* = 4.92, *SD* = 2.84).^5^ We also created a new variable for value reaffirmation with the remaining two restorative items (α = .86, *M* = 6.96, *SD* = 2.28).

##### Retributive justice

There was a significant main effect of condition on retributive justice, *F*(1, 237) = 354.13, *p* < .001, η^2^*p* = .60, 90% CI [.53, .65], such that participants were less supportive of retributive justice when the ingroup was the perpetrator (*M* = 2.74) than when it was the victim (*M* = 6.97). Importantly, the two-way interaction between ingroup role and identification was also significant (see Figure 2 in the Supplementary Document), *F*(1, 237) = 43.74, *p* < .001, η^2^*p* = .16, 90% CI [.09, .22]. Simple effects revealed that high identifiers supported significantly less retributive justice when the ingroup was the perpetrator (*M* = 2.26) rather than the victim (*M* = 7.88), *t*(237) = −17.80, *p* < .001. Low identifiers again exhibited the same pattern but to a much lesser degree (*M_perpetrator_* = 3.36, *M_victim_* = 6.09), *t*(237) = −8.52, *p* < .001. The main effect of identification did not reach significance, *F*(1, 237) = 1.32, *p* = .252, η^2^*p* = .01, 90% CI [.000, .03].

##### Value reaffirmation

The ingroup-role manipulation had a significant main effect on the extent to which participants supported value reaffirmation as an approach to restoring justice, *F*(1, 229) = 6.66, *p* = .011, η^2^*p* = .03, 90% CI [.004, .07]. Participants were less supportive of value reaffirmation when the ingroup was the perpetrator (*M* = 6.56) than when it was the victim (*M* = 7.32). The main effect of identification was marginally significant, *F*(1, 229) = 3.38, *p* = .067, η^2^*p* = .01, 90% CI [.000, .05], β = −.11. The interaction between condition and identification was not significant, *Fs*(1, 229) = .18, *p* = .671, η^2^*p* = .001, 90% CI [.000, .02].

#### Perpetrator perspective-taking as a moral disengagement mechanism

Consistent with our hypothesis, the extent to which participants took the perspective of ingroup perpetrators was highly and positively correlated with their use of exonerating cognitions, *r* = .77, *p* < .001 (ingroup-perpetrator condition: *r* = .66; ingroup-victim condition: *r* = .50).

**Figure 6.**
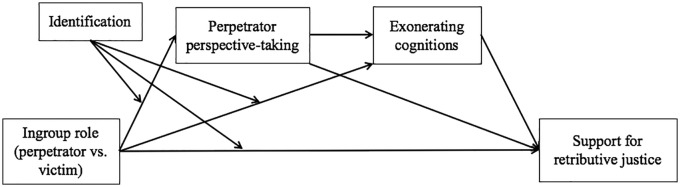
Moderated serial multiple mediator model (Process 3.0, Model 85) depicting the indirect effect of ingroup role on support for retributive justice through perpetrator perspective-taking and exonerating cognitions, moderated by national identification (Study 2).

**Figure 7.**
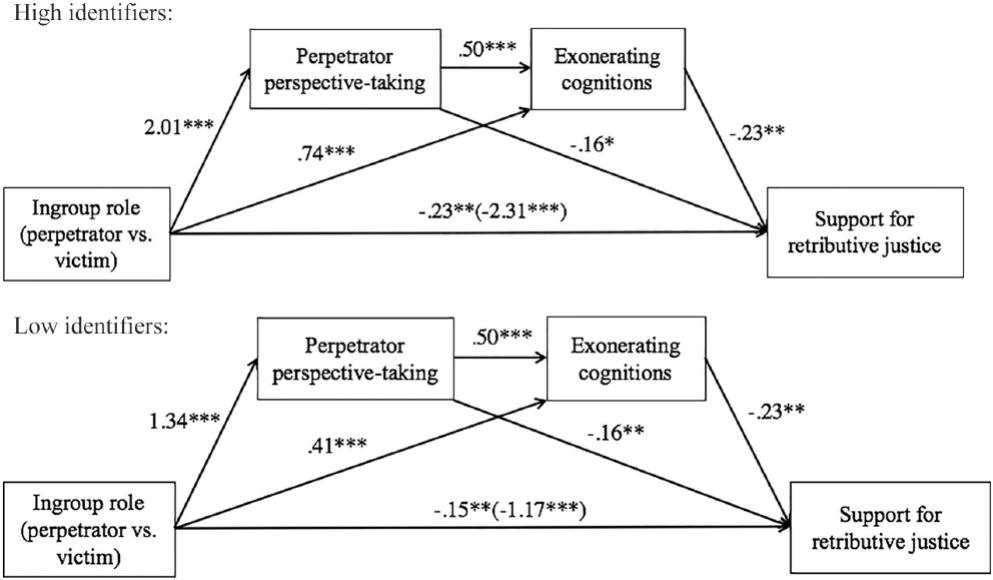
Moderated serial mediator models for high and low identifiers (Study 2). *Note.* All path coefficients are unstandardized.

To further test whether perpetrator perspective-taking serves a morally disengaging function, we conducted a moderated serial multiple mediation analysis, in which ingroup role was entered as the IV, retributive justice as the DV, perpetrator perspective-taking as the stage-one mediator, exonerating cognitions as the stage-two mediator, and identification as the moderator (Hayes, 2018, Model 85; see Figure 6 for the statistical model and Figure 7 for all path coefficients at high and low levels of identification). This model allowed us to test (a) the specific indirect effect through perpetrator perspective-taking, (b) the specific indirect effect through exonerating cognitions, and (c) the indirect effect through perspective-taking and exonerating cognitions in serial, thus taking into account the positive relationship between the two variables.

As predicted, the indirect effects of ingroup role on retributive justice through perpetrator perspective-taking and exonerating cognitions were both significant at high levels of identification (perspective-taking: *boot coefficient* = −.33, 95% CI [–.630, –.060]; exonerating cognitions: *boot coefficient* = −.17, 95% CI [–.368, –.034]). The indirect effects were also significant at low levels of identification, but to a much lesser degree (perspective-taking: *boot coefficient* = −.22, 95% CI [–.422, –.038]; exonerating cognitions: *boot coefficient* = −.09, 95% CI [–.222, –.013]). The moderated mediation indices were significant for perspective-taking, *boot coefficient* = −.06, 95% CI [–.140, –.005], and marginally so for exonerating cognitions, *boot coefficient* = −.04, 93% CI [–.102, –.001]). The analysis further revealed a significant indirect effect through perpetrator perspective-taking and exonerating cognitions as serial mediators at high levels of identification, *boot coefficient* = −.23, 95% CI [–.400, –.056], and at low levels of identification to a much lesser degree, *boot coefficient* = −.15, 95% CI [–.278, –.036]. Importantly, the overall moderated mediation index was significant, *boot coefficient* = −.04, 95% CI [–.086, –.005].

We also conducted the same moderated serial mediation analysis using support for value reaffirmation as the DV. Consistent with our hypothesis, no indirect effect through perpetrator perspective-taking or exonerating cognitions was significant, *boot coefficients* < .04, 95% CIs [–.440, .380]. Nor was the serial mediation through perspective-taking and exonerating cognitions significant, *boot coefficients* < .03, 95% CIs [–.217, .302].

### Discussion

Study 2 replicated the main findings of Study 1 in an entirely different intergroup context, showing that high identifiers were more likely to take the perspective of perpetrators when the ingroup had committed rather than suffered violence. Such perspective-taking, in turn, predicted their reduced support for retributive justice. Different from Study 1, the reactions of low identifiers in this study exhibited a similar pattern, but to a significantly lesser extent, than those of high identifiers. This finding was not surprising, however, given the long history of violent conflicts and political tensions between Israel and Arab countries. When perceived realistic threat is high between two parties, it is not surprising that even low identifiers would react defensively when reminded of past ingroup-committed violence against the adversarial group. Furthermore, the average level of identification was higher in the current study (*M* = 6.92) than in Study 1 (*M* = 5.57), suggesting that low identifiers in the Israeli sample might resemble more closely high rather than low identifiers in the American sample. Crucial to our hypothesis, our results showed that the defensive reactions of weakly identified Israelis were still significantly less strong than those of their highly identified counterparts.

Study 2 also extended Study 1 in two main ways. First, it provided evidence that taking the perspective of ingroup perpetrators indeed served as a moral disengagement mechanism at multiple levels. The mediational analysis revealed that perpetrator perspective-taking played a similar mediating role as the use of exonerating cognitions, which is typically seen as a moral disengagement mechanism (Roccas et al., 2006). The serial mediation further revealed an indirect effect of ingroup role on retributive justice through perpetrator perspective-taking and exonerating cognitions in sequence, with perspective-taking positively predicting exonerating cognitions, which in turn predicted reduced support for retributive justice. Given that both perpetrator perspective-taking and exonerating cognitions were measured mediators, the sequential relationship between the two should be interpreted with caution. Second, we showed that perpetrator perspective-taking explained the gap in perpetrator and victim group members’ support for retributive justice (operationalized as encompassing punishment, financial reparation, and apology), but not restorative justice (operationalized as reaffirmation of shared values).

Although Studies 1 and 2 provided convergent evidence for the morally disengaging function of perpetrator perspective-taking among high identifiers, the question remains whether perspective-taking *caused* the observed differences in support for retributive justice as a function of identification. Study 3 addressed this limitation, experimentally manipulating both the ingroup’s role in a conflict (victim or perpetrator) and perspective-taking with perpetrators.^6^

## Study 3

The primary goal of Study 3 was to provide further evidence for the causal chain depicted in Figure 1. By experimentally manipulating perpetrator perspective-taking, we aimed to establish its causal effects on support for retributive justice. We hypothesized that after being instructed to take the perspective of perpetrators (rather than an objective perspective), high identifiers would reduce their support for retributive justice when the ingroup has committed violence, but not when it has suffered violence. Low identifiers, in contrast, may not be affected by the perspective-taking manipulation in the same way that high identifiers should be. Although being instructed to take the perspective of ingroup perpetrators is consistent with high identifiers’ ingroup-defensive motivations and default reactions to ingroup transgression, it is not necessarily the case among low identifiers, as shown in Studies 1 and 2. Among Americans, taking the ingroup perpetrators’ perspective would in fact be contrary to low identifiers’ rather ingroup-critical reactions to ingroup transgression. According to the intention-based account of perspective-taking (Lucas et al., 2016), when people attribute malevolent intentions to the perpetrator, taking the perpetrator’s perspective actually increases their moral condemnation. As Study 3 was again conducted in the context of the U.S.–Iran conflict with American participants, we expected low identifiers to be even more critical of the ingroup, demanding more justice, after being instructed to take the perspective of ingroup perpetrators. In this study, we again measured both retributive and restorative justice to test whether adopting the perpetrators’ perspective would only reduce support for retributive, but not restorative, justice.

### Method

#### Participants

The sample consisted of 576 American adults recruited online through MTurk. Our screening of the data quality indicated that 85 participants did not pay sufficient attention to the news article, as indicated by their incorrect written summaries of the article and incorrect answers to manipulation check questions.^7^ In addition, 24 participants spent less than 7 minutes completing the survey.^8^ Six participants reported to have close friends or family members from Iran. Importantly, the altogether 112 excluded participants (19% of the total sample) were approximately evenly distributed across the study’s four conditions. A total of 464 participants were retained for subsequent analyses (58% women; age *M* = 39, *SD* = 12.89).

#### Procedure

We tested the causal effect of perpetrator perspective-taking on support for justice in a 2 (ingroup role: victim vs. perpetrator) × 2 (perpetrator perspective-taking manipulation: perspective-taking vs. objective) experimental design. The ingroup-role manipulation was very similar to the one in Study 1, where participants read a short news excerpt describing prisoner abuses either committed or suffered by Americans vis-à-vis Iranians. The news excerpts were adapted from Study 1 with some minor modifications, including the shortened length and the individualization of responsibility by providing the names of one perpetrator (“Michael Smith”) and his victim (“Amir Mohsen”). Both the perpetrator and the victim were given male names that are relatively common in either Western or Middle Eastern countries, depending on condition.

To manipulate perspective-taking, we used instructions very similar to those employed by Batson and colleagues (1997). Prior to reading the news excerpt, all participants were told that they were about to read an excerpt from a news story about an [American/Iranian] officer in a military prison at the border between Iran and Afghanistan. In the *perspective-taking* condition, participants were then instructed to “take the perspective of the [American/Iranian] military officer described in the story” and also to “imagine what the officer was thinking and how he was feeling.” In the *objective* condition, participants were instructed to “take an objective perspective towards the acts of the [American/Iranian] military officer” and to “not get caught up in what the officer thinks and how he feels; just remain objective and detached.” Afterwards, participants completed manipulation checks regarding the ingroup’s role in the conflict and summarized the news excerpt in their own words. To reinforce the perspective-taking manipulation, participants were instructed to summarize the excerpt either from the perspective of the military officer (perspective-taking condition) or remain objective in their summaries (objective condition). Afterwards, participants completed measures of perspective-taking with perpetrators, support for retributive and restorative justice, and ingroup identification.

#### Materials

##### Perspective-taking manipulation check

To assess the effectiveness of the perspective-taking manipulation, four items measured the extent to which participants took the perspective of the perpetrator, *Michael Smith* or *Amir Mohsen* (e.g., “*I tried to see things from [Michael Smith’s/Amir Mohsen’s] point of view*.”). The items were worded slightly differently from those in Studies 1 and 2 because we adapted them to anchor to an identifiable individual perpetrator rather than the perpetrator group.

Support for retributive and restorative justice and ingroup attachment and glorification were measured using the same items as in the previous studies.

### Results

#### Perspective-taking manipulation check

To assess the effectiveness of the perspective-taking manipulation, we first subjected participants’ scores on the perpetrator perspective-taking measure (α = .80, *M* = 4.40, *SD* = 2.02) to a generalized linear model (GLM) with the perspective-taking and ingroup-role manipulations as IVs and identification as a continuous moderating variable. The main effect of the perspective-taking manipulation was significant, such that participants in the perspective-taking condition took more perspective of the perpetrator (*M* = 5.14) than those in the objective condition (*M* = 3.80), regardless of the perpetrator’s group identity, *F*(1, 456) = 60.78, *p* < .001, η^2^*p* = .12, 90% CI [.07, .16].

Replicating the findings in Studies 1 and 2, the analysis also revealed a significant two-way interaction between ingroup role and identification, *F*(1, 456) = 20.39, *p* < .001, η^2^*p* = .04, 90% CI [.02, .08]. Simple effects revealed that high identifiers took significantly more perspective of the perpetrator when the ingroup committed (*M* = 5.26) rather than suffered violence (*M* = 4.12), *t*(456) = 4.73, *p* < .001, whereas low identifiers exhibited the opposite pattern (*M_perpetrator_* = 4.00, *M_victim_* = 4.47), *t*(456) = −1.84, *p* = .067.

Overall, these results indicate that the perspective-taking manipulation was successful and did not interact with the ingroup-role manipulation or identification. Other effects that were not central to our hypotheses are reported in the Supplementary Document.

### The Joint Effects of Ingroup Role, Perspective-Taking, and Identification

#### Support for justice

Participants’ support for retributive justice was again strongly correlated with their support for restorative justice, *r* = .65, *p* < .001. An EFA (using parallel analysis and a scree plot) yielded the same factor solution as in Study 2, suggesting that American participants also construed financial reparations and apologies as punitive, similar to retributive justice. We thus again created a new retributive justice variable, including both the original five retributive items and the three restorative items (α = .93, *M* = 5.77, *SD* = 1.92),^9^ as well as a new restorative justice variable, including the two items concerning value reaffirmation (α = .90, *M* = 6.95, *SD* = 1.89).

##### Retributive justice

**Figure 8.**
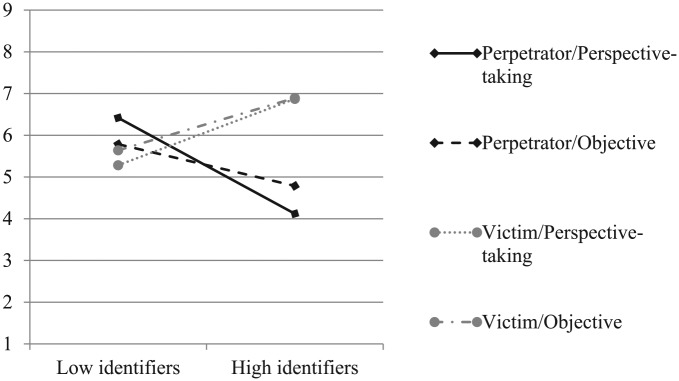
Support for retributive justice as a function of ingroup’s role (perpetrator vs. victim), perspective-taking with perpetrators (perspective-taking vs. objective), and national identification (Study 3).

Support for retributive justice was submitted to a GLM with the manipulations of perspective-taking and ingroup role as IVs and identification as a continuous moderator. Consistent with our hypothesis that taking the perpetrator’s perspective would reduce high (but not low) identifiers’ retributive justice support when the ingroup was the perpetrator (but not the victim) in the conflict, we obtained a significant three-way interaction of identification by the perspective-taking manipulation and ingroup role (see Figure 8), *F*(1, 456) = 6.19, *p* = .013, η^2^*p* = .01, 90% CI [.002, .04].

When the ingroup committed violence, taking the ingroup perpetrator’s perspective significantly reduced high identifiers’ support for retributive justice (*M* = 4.12), compared to taking an objective perspective (*M* = 4.79), *t*(456) = −2.09, *p* = .037. Low identifiers, by contrast, exhibited the opposite pattern. Taking the ingroup perpetrator’s perspective (*M* = 6.42) increased their support for retributive justice, compared to taking an objective perspective (*M* = 5.79), *t*(456) = 1.97, *p* = .050. When the ingroup suffered violence, however, being instructed to take the perspective of outgroup perpetrators did not significantly alter either high (*M_perspective-taking_* = 6.87, *M_objective_* = 6.90) or low (*M_perspective-taking_* = 5.28, *M_objective_* = 5.64) identifiers’ support for justice, *ts*(456) < –1.05, *ps* > .290.

As in previous studies, the analysis also revealed a significant two-way interaction between ingroup role and identification, *F*(1, 456) = 87.31, *p* < .001, η^2^*p* = .16, 90% CI [.11, .21]. High identifiers supported significantly less retributive justice when the ingroup was the perpetrator (*M* = 4.45) rather than the victim (*M* = 6.88), *t*(456) = −10.94, *p* < .001, whereas low identifiers supported significantly more retributive justice when the ingroup was the perpetrator (*M* = 6.10) rather than the victim (*M* = 5.46), *t*(456) = 2.73, *p* = .007.

##### Value reaffirmation

We conducted the same analysis with value reaffirmation as the DV. As expected, neither the three-way interaction of identification by perspective-taking and ingroup role nor the two-way interaction between identification and ingroup role was significant, *Fs*(1, 456) < .40, *ps* > .540, η^2^*_p_s* < .001.

Other effects that were not central to the hypothesis are reported in the Supplementary Document.

### Discussion

Study 3 experimentally manipulated both perpetrator perspective-taking and participants’ ingroup’s role as perpetrator or victim in the conflict. Consistent with our predictions, taking the perpetrator’s perspective reduced high identifiers’ support for retributive justice when the ingroup has committed harm against another group. Low identifiers, by contrast, reacted in the opposite manner, demanding more justice after being instructed to take the perspective of ingroup perpetrators. Low identifiers’ reactions were consistent with the intention-based account of perspective-taking (Lucas et al., 2016). As low levels of identification are often associated with ingroup-critical attitudes when faced with ingroup-committed wrongdoings (e.g., Leidner & Castano, 2012), focusing attention toward ingroup perpetrators’ thoughts and feelings might have amplified their initially critical evaluation of the wrongdoing. This finding also highlights the distinction between measured or self-reported perspective-taking and manipulated perspective-taking. Although self-reported perspective-taking with the ingroup perpetrators predicted less support for retributive justice regardless of identification (as shown in Studies 1 and 2), manipulated perspective-taking had divergent effects on high and low identifiers’ support for retributive justice.

When the ingroup was the victim of intergroup transgressions, on the contrary, taking the perspective of (outgroup) perpetrators did not reduce justice support among high identifiers, further suggesting that perspective-taking serves a morally disengaging function when the perpetrator belongs to the ingroup. Study 3 thus provided additional evidence that perspective-taking can be a motivated cognitive process in intergroup conflicts, and that perpetrator perspective-taking is a causal mechanism underlying the effects of ingroup’s role in a conflict on group members’ support for justice. Moreover, Study 3 complemented the finding in Study 2 and causally showed that perspective-taking with ingroup perpetrators reduced support for punitive justice measures, but not support for efforts to reaffirm shared values between perpetrator and victim groups.

## General Discussion

In two intergroup contexts, we investigated perpetrator perspective-taking in the aftermath of intergroup transgressions and its implications for retributive justice among both victim and perpetrator group members. Among Americans who strongly identified with their country, reminders of ingroup perpetration (as opposed to victimization) in the conflict between the United States and Iran increased their perspective-taking with the perpetrators. Our mediation analyses further showed that adopting the perspective of ingroup perpetrators explained high identifiers’ reduced support for justice in the form of perpetrator punishment (Study 1). Study 2 replicated these findings in the context of a historical conflict between Israel and Syria, and extended them to other justice mechanisms, namely apology and financial reparation, but not efforts to reaffirm the shared values between groups. Furthermore, Study 2 demonstrated the morally disengaging function of perpetrator perspective-taking, particularly in the form of using exonerating cognitions to minimize the ingroup’s moral transgressions. Finally, Study 3 presented causal evidence for the effect of perpetrator perspective-taking on support for retributive justice.

By examining participants’ responses to ingroup perpetration and victimization, our methodological approach considered victim and perpetrator perspectives in tandem. The current work thus complements the previous research that has mostly focused on only one of these perspectives and provides a rather comprehensive outlook on the role of perspective-taking in the context of intergroup transgressions. It also brings together two previously separated lines of research—namely, perspective-taking and moral disengagement—and demonstrates for the first time that taking the perspective of ingroup perpetrators can play a similar role as moral disengagement, leading to reduced support for retributive justice.

### Perspective-Taking as Motivated Cognition

Our findings highlight the motivated nature of perspective-taking in the context of intergroup relations. Substantial research has demonstrated that when confronted with ingroup-committed wrongdoings, people are motivated to execute various affective and cognitive strategies that allow them to morally disengage from the wrongdoings (e.g., Aquino, Reed, Thau, & Freeman, 2007; Bandura, 1999). Unlike the more commonly studied moral disengagement strategies such as victim dehumanization and moral justification, adopting the perspective of ingroup perpetrators does not imply an explicit intention to disengage from moral transgressions. When motivated by ingroup-committed (rather than suffered) violence; however, this basic cognitive process predicted the use of exonerating cognitions and subsequently led to reduced support for retributive justice. The present research thus also contributes to the existing literature by showing that even without any explicit intention to morally disengage, cognitively appraising the intergroup situation from the perpetrators’ perspective can take on a morally disengaging function when the perpetrators belong to one’s ingroup. In other words, attempts to understand ingroup perpetrators can be a slippery slope to injustice and prevent accountability of the ingroup.

The observed moderating effect of identification adds another layer of complexity to the motivational component of perspective-taking in response to intergroup conflict. Consistent with prior research on ingroup identification in general (Branscombe et al., 1999; Doosje et al., 1995) and ingroup glorification in particular (Bilali, 2013; Leidner & Castano, 2012; Leidner et al., 2010; Li, Leidner, Euh, & Choi, 2016; Roccas et al., 2006), we demonstrated that members of the perpetrator group were motivated to adopt the perpetrators’ perspective only to the extent that they strongly identified with their own group. Moreover, while we successfully induced perspective-taking with perpetrators among both high and low identifiers, it only reduced justice support in response to ingroup (but not outgroup) atrocities among high identifiers, while increasing justice support among low identifiers. Therefore, perpetrator perspective-taking and its exonerating function are essentially motivated by the basic psychological need to defend against threats to the moral image of the ingroup.

It should also be noted that in the current studies, using glorification or attachment as the moderator while controlling for the other yielded similar results as using overall identification as the moderator. Previous research has produced mixed findings regarding the distinction between glorification and attachment. Although most of the research has demonstrated the detrimental role of glorification—but not attachment—in intergroup conflicts (e.g., Leidner & Castano, 2012; Leidner et al., 2010; Li et al., 2016; Roccas et al., 2006), recent work showed that glorification and attachment moderated conflict attitudes in similar ways (Shuman, Johnson, Saguy, & Halperin, 2018). Future research should thus investigate when and under what conditions these two dimensions of identification play similar or distinct roles.

### The Pitfalls of Understanding Evil

The present research also bears on an intriguing philosophical and practical question in responding to acts of evil: should we attempt to understand those who carry out these acts, to view things through their eyes, and to imagine their thoughts, feelings, and intentions? There certainly are benefits of understanding perpetrators and their actions, especially for victims who are struggling to forgive and move on (Takaku, 2001; Takaku et al., 2001). For observers of transgressions, however, it has been cautioned that understanding the perspective of perpetrators might carry the moral risk of diminishing the severity of their crimes (Baumeister, 1997). Indeed, very few people who have committed severe moral transgressions actually see their actions as evil. Understanding the perpetrators on their own terms, therefore, can come at the cost of impartial moral judgments, especially when driven by defensive motivations. By showing that high identifiers are particularly prone to adopting the perspective of ingroup perpetrators and hence seeing their crimes as less punishable, our findings offer empirical evidence for this risk, and establish a boundary condition for its occurrence (i.e., levels of ingroup identification).

### Limitations and Future Directions

Several limitations of the current research should also be noted. First, due to our focus on parties directly involved in acts of moral transgression, it will be important for future research to further investigate perpetrator perspective-taking and its implications from the perspective of neutral third parties of intergroup transgressions. Second, the long-standing conflict between the United States and Iran, which could be amplified by the reminder of Iran’s nuclear program in the manipulation, might have triggered perceptions of realistic threat even in the U.S.-perpetration condition. Such threat perceptions might have contributed to high identifiers’ rather defensive reactions to the immoral acts committed by the ingroup. The same psychological processes might have also contributed to the defensive reactions among both highly and weakly identified Israeli participants. While our findings are limited by the existing relationships between the involved parties, such macro-level sociopolitical contexts are inevitable in the study of real, large-scale intergroup conflicts. Finally, our cognitive rather than affective operationalization of “understanding perpetrators” does not address the emotional aspects of understanding moral transgressions. Empathizing with perpetrators, for example, may also evoke intense emotional responses (Davis, 1980) that can further cloud observers’ moral judgments and eventually obstruct the pursuit of justice. Therefore, future work should examine affective and cognitive processes in tandem.

## Conclusion

This research suggests that in response to intergroup violence, high identifiers are more likely to take the perspective of perpetrators when their own group has committed rather than suffered violence. Such engagement in perpetrator perspective-taking further serves the function to facilitate exonerating appraisals of the transgressions, leading to high identifiers’ reduced support for retributive justice. These findings uncovered the undesirable consequences of understanding acts of evil in the context of intergroup conflict and can have important implications for the restoration of justice in postconflict societies.

## Supplemental Material

Li_OnlineAppendix – Supplemental material for Stepping into Perpetrators’ Shoes: How Ingroup Transgressions and Victimization Shape Support for Retributive Justice through Perspective-Taking With Perpetrators Click here for additional data file. Supplemental material, Li_OnlineAppendix for Stepping into Perpetrators’ Shoes: How Ingroup Transgressions and Victimization Shape Support for Retributive Justice through Perspective-Taking With Perpetrators by Mengyao Li, Bernhard Leidner and Silvia Fernandez-Campos in Personality and Social Psychology Bulletin

Perpetrator_Perspective-taking_Supplementary_Document_R2 – Supplemental material for Stepping into Perpetrators’ Shoes: How Ingroup Transgressions and Victimization Shape Support for Retributive Justice through Perspective-Taking With Perpetrators Click here for additional data file. Supplemental material, Perpetrator_Perspective-taking_Supplementary_Document_R2 for Stepping into Perpetrators’ Shoes: How Ingroup Transgressions and Victimization Shape Support for Retributive Justice through Perspective-Taking With Perpetrators by Mengyao Li, Bernhard Leidner and Silvia Fernandez-Campos in Personality and Social Psychology Bulletin

## Notes

1. Analyses from this dataset that are not relevant to the hypotheses considered here were previously reported in a different article (Li, Leidner, Petrović, Orazani, & Rad, 2018). Although the design of the study was the same, the earlier article focused on the underlying mechanisms of victim—not perpetrator—group members’ responses to intergroup conflict and did not test the mechanism underlying perpetrator group members’ responses (i.e., perspective-taking with perpetrators) that is the focus of this article.

2. We conducted the same analyses in all following studies. As identification was not significantly affected by the experimental manipulations, we only report the results in detail in the Supplementary Document.

3. Following the recommendations by Lakens (2013) and Steiger (2004), we report 90% CIs for partial eta squared throughout the article. Given that *F* tests are one-sided and η^2^*p* cannot be negative, computing 90% CIs is more appropriate and equivalent to a significance level of α = .05.

4. As in Study 1, using glorification or attachment as a moderator while controlling for the other yielded similar patterns of results.

5. We also conducted analyses with the original retributive and restorative justice scales as the DVs, and both analyses yielded significant two-way interactions between identification and ingroup role.

6. We conducted an additional study intended to replicate the findings from Studies 1 and 2, as well as to examine the perspective of a third-party group (in addition to the perpetrator vs. victim perspective). The context of this study was similar to that of Study 1. The primary results revealed that in conditions where the participants’ ingroup (the United States) was described as the perpetrator or the victim (as in Studies 1 and 2), high identifiers exhibited the same pattern as in Studies 1 and 2, such that they increased their perspective-taking with the perpetrator and reduced their support for retributive justice when the ingroup had committed rather than suffered violence. The full study design and the relevant results are reported in detail in the Supplementary Document.

7. Because participants in this study read a short news excerpt in a text format rather than an actual news article, we did not record their reading time.

8. The average amount of time that participants took to complete the survey was 13.4 minutes, and we used half of the average time as the cutoff. The results remained unchanged when including these 24 participants.

9. Using the original retributive and restorative justice scales as DVs yielded significant and marginally significant three-way interactions of identification by perspective-taking and ingroup role, respectively.
