# Supplementary material for: Stepping into Perpetrators’ Shoes: How Ingroup Transgressions and Victimization Shape Support for Retributive Justice through Perspective-Taking With Perpetrators
Source: Pers Soc Psychol Bull. 2019 Jun 27;46(3):424–38. doi: 10.1177/0146167219858652 (PMC6993134; doi:10.1177/0146167219858652)
Supplement: Li_OnlineAppendix – Supplemental material for Stepping into Perpetrators’ Shoes: How Ingroup Transgressions and Victimization Shape Support for Retributive Justice through Perspective-Taking With Perpetrators [file Li_OnlineAppendix.pdf]

Manipulation Materials

Study 1
Ingroup-perpetrator condition:

Title: Investigation Revealed American Abuse of Iranian Prisoners

A recent investigation conducted by the Committee for Justice in the Middle East (CJME), revealed cases of serious abuse of Iranian prisoners at a secret prison at the Iran-Afghan border that is under the control of the United States Armed Forces.

In recent years, tensions have mounted between the United States and Iran due to increasing concerns about Iran's nuclear program. In 2011, the two countries elevated the belligerent tone between them over an Iranian vow to close the Strait of Hormuz, a vital Middle East waterway for oil tanker traffic, if the U.S. attempted to make good on its threat to stifle Iran's petroleum exports.

With the escalation of the conflict between Iran and the United States, a number of Iranians working at the border were detained and interrogated by the U.S. military.

Brigadier General Adam McLaughlin, formerly in charge of the prison, provided information in an interview with a CJME official investigating the abuses at the prison. The transcript of this May 2012 interview was among hundreds of pages of papers obtained by CJME.

The investigation provides detailed descriptions of three cases of severe maltreatment of Iranian detainees at the prison.

One of the detainees was placed in solitary confinement with his eyes blindfolded and his arms tied behind his back. During an interrogation session, several American military officers suffocated him with a bag until he lost consciousness and woke him with an electric shock to his genitals.

Another detainee, a 21-year old man, died after three American soldiers employed a sleep deprivation technique for 72 hours while beating him numerous times on the head and body. His head was so badly damaged that identification was possible only through DNA samples retrieved from his home.

The third detainee, who was arrested in March 2012, endured a horrific beating while being hung upside down. The interrogation lasted for eight hours without interruption. When he was brought back to his cell, another detainee who had been a doctor prior to his arrest noticed swelling above his fellow detainee's liver and suspected internal bleeding.

"American security officials whipped detainees with heavy cables, pulled out finger and toenails, burned them with acid and cigarettes, and smashed their teeth," said Kenneth Stork, the deputy director of CJME. "The evidence we found suggests that such interrogation techniques were the norm at the prison." Mr. Stork called for a thorough investigation of all officials and individuals "responsible for this systematic brutality."

Ingroup-victim condition:

Title: Investigation Revealed Iranian Abuse of American Prisoners

A recent investigation conducted by the Committee for Justice in the Middle East (CJME), reported cases of serious abuse of American prisoners at a secret prison at the Iran-Afghan border that is under the control of the Iranian Revolutionary Guards, a branch of Iran's military founded after the Islamic Revolution of 1979.

In recent years, tensions have mounted between the United States and Iran due to increasing concerns about Iran's nuclear program. In 2011, the two countries elevated the belligerent tone between them over an Iranian vow to close the Strait of Hormuz, a vital Middle East waterway for oil tanker traffic, if the U.S. attempted to make good on its threat to stifle Iran's petroleum exports.

With the escalation of the conflict between Iran and the United States, a number of Americans working at the Iran-Afghan border were detained and interrogated by the Revolutionary Guards.

Brigadier General Ali Fazli, formerly in charge of the prison, provided information in an interview with a CJME official investigating the abuses at the prison. The transcript of this May 2012 interview was among hundreds of pages of papers obtained by CJME.

The investigation provides detailed descriptions of three cases of severe maltreatment of American detainees at the prison.

One of the detainees was placed in solitary confinement with his eyes blindfolded and his arms tied behind his back. During an interrogation session, several Iranian military officers suffocated him with a plastic bag until he lost consciousness and woke him with an electric shock to his genitals.

Another detainee, a 21-year-old man, died after three Iranian soldiers employed a sleep deprivation technique for 72 hours while beating him numerous times on the head and body. His head was so badly damaged that identification was possible only through DNA samples retrieved from his home.

The third detainee, who was arrested in March 2012, endured a horrific beating while being hung upside down. The interrogation lasted for eight hours without interruption. When he was brought back to his cell, another detainee who had been a doctor prior to his arrest noticed swelling above his fellow detainee's liver and suspected internal bleeding.

"Iranian security officials whipped detainees with heavy cables, pulled out finger and toenails, burned them with acid and cigarettes, and smashed their teeth," said Kenneth Stork, the deputy director of CJME. "The evidence we found suggests that such interrogation techniques were the norm at the prison." Mr. Stork called for a thorough investigation of all officials and individuals "responsible for this systematic brutality."

Study 2:

Ingroup-perpetrator condition:

Title: Recently declassified documents reveal: Israeli soldiers tortured Syrian civilians during Yom Kippur War

Recently declassified documents reveal severe cases of torture of Syrian civilians by Israeli soldiers that took place during the 1973 Yom Kippur War.

Since the surprise attack by Syria and Egypt on Israel on October 6, 1973, the Israeli Defense Forces (IDF) had launched a series of counterattacks against the Arab coalition. As part of the war effort, the Israeli military established a special secret unit whose primary job was to gather Syrian intelligence. Located in a secret facility near the Syrian-Israeli border, this unit arrested hundreds of Syrians from nearby towns and villages. According to the released documents, these arrests involved torture and severe mistreatment of detainees, many of whom were clearly innocent civilians whose participation in any anti-Israel activity was highly questionable. The documents describe three specific instances of abuse and torture of Syrian civilians.

In one case, the Syrian detainee’s arms and legs were tied and he was blindfolded, and then a group of Israeli soldiers placed a plastic bag over his head until he lost consciousness. The way to retrieve his conscious was by administering electric shocks to his genitals. In another case, a Syrian detainee died after three soldiers denied him food and water for three days, and beat him repeatedly on the head and all over his body. The third case describes a Syrian who was arrested, hung upside down, and severely beaten for eight hours. When he returned to his cell, another prisoner who was a doctor examined him and reported that he had signs of internal bleeding and liver damage.

The documents conclude that "it is abundantly clear that at the facility in question, Israeli forces severely tortured Syrian civilians during the war, and that this treatment was the norm in this facility."

Study 2:

Ingroup-victim condition:

Title: Recently declassified documents reveal: Syrian soldiers tortured Israeli civilians during Yom Kippur War

Recently declassified documents reveal severe cases of torture of Israeli civilians by Syrian soldiers that took place during the 1973 Yom Kippur War.

Since the surprise attack by Syria and Egypt on Israel on October 6, 1973, the Israeli Defense Forces (IDF) had launched a series of counterattacks against the Arab coalition. As part of the war effort, the Syrian Armed Forces established a special secret unit whose job was to gather Israeli intelligence. Located in a secret facility near the Syrian-Israeli border, this unit arrested hundreds of Israelis from nearby towns and villages. According to the released documents, these arrests involved torture and severe mistreatment of detainees, many of whom are clearly innocent civilians whose participation in any anti-Syrian activity is highly questionable. The documents describe three specific instances of abuse and torture of Israeli civilians.

In one case, the Israeli detainee's arms and legs were tied and he was blindfolded, and then a group of Syrian soldiers placed a plastic bag over his head until he lost consciousness. The way to retrieve his conscious was by administering electric shocks to his genitals. In another case, an Israeli detainee died after three soldiers denied him food and water for three days, and beat him repeatedly on the head and all over his body. The third case describes an Israeli who was arrested, hung upside down, and severely beaten for eight hours. When he returned to his cell, another prisoner who was a doctor examined him and reported that he had signs of internal bleeding and liver damage.

The documents conclude that "it is abundantly clear that at the facility in question, Syrian forces severely tortured Israeli civilians during the war, and that this treatment was the norm in this facility."

Study 3
Perspective-taking condition:

On the next page, you will read an excerpt from a news story about an American/Iranian officer in a military prison at the border between Iran and Afghanistan.

While you are reading, try to take the perspective of this American/Iranian military officer described in the story. That is, try to put yourself in the officer's shoes. Imagine what the officer was thinking and how he was feeling.

Objective condition:

On the next page, you will read an excerpt from a news story about an American/Iranian officer in a military prison at the border between Iran and Afghanistan.

While you are reading, try to take an objective perspective towards the acts of this American/Iranian military officer. That is, try to **NOT** get caught up in what the officer thinks and how he feels; just remain objective and detached.

Study 3
Ingroup-victim condition:

Investigation Revealed Iranian Abuse of American Prisoners: The Case of Officer Amir Mohsen

In recent years, news has surfaced that the Iranian military interrogated Americans detained in a secret military prison at the border between Iran and Afghanistan. Some say these interrogations amounted to serious prisoner abuse.

Amir Mohsen was one of the Iranian military officers carrying out the interrogation of American detainees. Officer Mohsen was allegedly responsible for the death of a 21-year-old detainee. During the interrogation session, Officer Mohsen employed a sleep deprivation technique for 72 hours while beating the detainee numerous times on the head and body.

The evidence collected so far suggests that such interrogation techniques were the norm at the prison. The Amir Mohsen case was one of the many that are currently under investigation.

Ingroup-perpetrator condition:

Investigation Revealed American Abuse of Iranian Prisoners: The Case of Officer Michael Smith

In recent years, news has surfaced that the American military interrogated Iranians detained in a secret military prison at the border between Iran and Afghanistan. Some say these interrogations amounted to serious prisoner abuse.

Michael Smith was one of the American military officers carrying out the interrogation of Iranian detainees. Officer Smith was allegedly responsible for the death of a 21-year-old detainee. During the interrogation session, Officer Smith employed a sleep deprivation technique for 72 hours while beating the detainee numerous times on the head and body.

The evidence collected so far suggests that such interrogation techniques were the norm at the prison. The Michael Smith case was one of the many that are currently under investigation.

Manipulation Checks:

The remainder of the survey will focus on your perception of the events reported in the newspaper article you have just read. First, we have a few questions regarding your understanding of the newspaper article. For each of the questions below, please choose only ONE answer by checking one of the circles on the left.

Studies 1 and 3:

Who were the victims in the events you have just read about in the news report?

- ❍ Americans
- ❍ Iranians
- ❍ Both sides were victims and perpetrators
- ❍ Do not know

Who were the perpetrators in the events you have just read about in the news report?

- ❍ Americans
- ❍ Iranians
- ❍ Both sides were victims and perpetrators
- ❍ Do not know

Study 2:

Who were the victims in the events you have just read about in the news report?

- Israelis
- Syrians
- Both sides were victims and perpetrators
- Do not know

Who were the perpetrators in the events you have just read about in the news report?

- Israelis
- Syrians
- Both sides were victims and perpetrators
- Do not know

Next, we would like you to write a brief summary of what you have read about in the beginning of this study. The goal is that a third person who has not read the document will be able to understand its contents by just reading your summary. Please address specifically the content of the conflict.

***Dependent Measures* [1]**

Perspective taking with perpetrators:

Studies 1 & 2

1. I tried to understand these [perpetrator group] soldiers better by imagining how things looked from their perspective.
2. I believe that there are two sides to the actions of these [perpetrator group] soldiers and tried to look at both of these sides.
3. I tried to "put myself in the shoes" of these [perpetrator group] soldiers.
4. Before criticizing these [perpetrator group] soldiers, I tried to imagine how I would feel if I were in their place.
5. I found it difficult to see things from these [perpetrator group] soldiers' point of view.

Study 3

1. I tried to see things from Michael Smith's (Amir Mohsen's) point of view.
2. It was very easy for me to imagine how Michael Smith (Amir Mohsen) was feeling.
3. I could imagine myself as Michael Smith (Amir Mohsen).
4. It was very difficult for me to understand what Michael Smith (Amir Mohsen) was thinking in the scenario described.

[1] All items were measured on 9-point scales in Study 1 and 2 (1 = strongly disagree; 6 or 9 = strongly agree).

Exonerating Cognitions (Study 2)

We are interested in how you feel about the actions of the [perpetrator group] military as described in the news article.

1. The language that the news article used to describe the actions of the [perpetrator group] military is too harsh.

2. The actions of the [perpetrator group] military are understandable, given the circumstances during this time period.

3. The [victim group] detainees deserved the treatment they received.

4. While the events in the news excerpt were unfortunate, such things cannot always be prevented when two countries are at war.

5. Sometimes it is unavoidable to do something bad for the sake of the greater good, and that is what happened in the situations described in the news report.

Support for Justice:

We are interested in how, in your opinion, justice needs to be restored in the conflict between [Iran and the United States/Israel and Syria]. There are no right or wrong answers.

Please indicate the extent to which you agree or disagree with the following statements.

Retributive justice:

1. To fully restore justice, [perpetrator group] needs to be punished for its military's actions described in the news report.
2. For justice to be fully reinstated, punitive measures should be adopted to address the military actions carried out by [perpetrator group].
3. To fully reinstate justice, the Human Rights Court needs to prosecute [perpetrator group] for its role in the events described in the news report.
4. To fully restore justice, [perpetrator group] needs to receive punishment for the events described in the news report.
5. Justice is only fully served when there are punitive measures against [perpetrator group] for its actions described in the news report.

Restorative justice:

1. To restore justice, [perpetrator group] and [victim group] need to agree on rules of a peaceful world.
2. For justice to be reinstated, [perpetrator group] and [victim group] need to agree on ethical values that should not be violated.
3. Without a sincere apology from [perpetrator group] for having acted wrongly, the injustice is not completely restored.
4. To restore justice, the [victim group] detainees and their family members need to receive financial compensation from [perpetrator group] for what happened in the prison.
5. For justice to be reinstated, [perpetrator group] needs to express remorse to the [victim group] detainees and their family members for what happened in the prison.

National Attachment:

We are interested in how you perceive yourself as an American/Israeli citizen. Again, there are no correct or incorrect answers.

1. I love the United States/Israel.
2. Being American/Israeli is an important part of my identity.
3. It is important for me to view myself as an American/Israeli.
4. It is important for me to contribute to my nation.
5. I am strongly committed to the United States/Israel.
6. It is important for me that everyone sees me as an American/Israeli.
7. It is important for me to help my country.
8. When I talk about Americans/Israelis I usually say "we" rather than "they."

National Glorification:

Please report your opinion on the role of the United States/Israel in the world. Please answer all questions. Again, there are no correct or incorrect answers.

1. The U.S./Israeli Armed Forces is the best army in the world.
2. It is disloyal for Americans/Israelis to criticize the United States/Israel.
3. One of the important things that we have to teach our children is to respect the leaders of our nation.
4. Other nations can learn a lot from us.
5. Relative to other nations, we are a very moral nation.
6. There is generally a good reason for every rule and regulation made by our national authorities.
7. In today's world, the only way to know what to do is to rely on the leaders of our nation.
8. The U.S. /Israel is better than other nations in all respects.
