# Supplementary material for: Stepping into Perpetrators’ Shoes: How Ingroup Transgressions and Victimization Shape Support for Retributive Justice through Perspective-Taking With Perpetrators
Source: Pers Soc Psychol Bull. 2019 Jun 27;46(3):424–38. doi: 10.1177/0146167219858652 (PMC6993134; doi:10.1177/0146167219858652)
Supplement: Perpetrator_Perspective-taking_Supplementary_Document_R2 – Supplemental material for Stepping into Perpetrators’ Shoes: How Ingroup Transgressions and Victimization Shape Support for Retributive Justice through Perspective-Taking With Perpetrators [file Perpetrator_Perspective-taking_Supplementary_Document_R2.pdf]

Supplementary Document

Additional Results

Study 2:

Ingroup identification. The ingroup role manipulation did not significantly affect levels of ingroup identification, $F(1, 243) = 0.46$, $p = .497$, $\eta^2_p < .001$.

Study 3

Ingroup identification. Neither did the ingroup role nor the perspective-taking manipulation significantly affect levels of identification, $Fs(1, 460) < 2.00$, $ps > .165$, η^2_ps $< .01$.

Perspective-taking manipulation check. There was also a marginally significant main effect of ingroup role, such that participants engaged in somewhat more perpetrator perspective-taking when their ingroup committed ($M = 4.63$) rather than suffered violence ($M = 4.17$), $F(1, 456) = 3.80$, $p = .052$, $\eta^2_p = .01$, *90% CI* [.00, .03]. The main effect of identification was also significant, $F(1, 456) = 6.46$, $p = .011$, $\eta^2_p = .01$, *90% CI* [.002, .04]. No other effects reached significance, $Fs(1, 456) < 1.55$, $ps > .220$, $\eta^2_ps < .01$.

Retributive justice. The analysis also revealed a significant main effect of ingroup role, $F(1, 456) = 31.62$, $p < .001$, $\eta^2_p = .06$, *90% CI* [.03, .10], such that participants were less supportive of retributive justice when the ingroup was the perpetrator ($M = 5.24$) than the victim ($M = 6.20$). No other effects reached significance, $Fs(1, 456) < 2.16$, $ps > .140$, η^2_ps $< .005$.

Value re-affirmation. The analysis revealed a significant main effect of ingroup role, such that participants were less in favor of value re-affirmation as a means to restore justice when the ingroup was the perpetrator ($M = 6.60$) rather than the victim ($M = 7.30$), $F(1, 456) = 16.16$, $p < .001$, $\eta^2_p = .03$, *90% CI* [.01, .06]. The interaction between identification and the perspective-taking manipulation was marginally significant, $F(1, 456) = 2.97$, $p = .086$, η^2_p

= .01, *90% CI* [.00, .02]. None of the simple effects, however, reached significance, $ts(456) < 1.60$, $ps > .120$. No other effects was significant, $Fs(1, 456) < 1.60$, $ps > .210$, $\eta^2_{p}s < .01$.

Additional Study

Method

Participants. The sample consisted of 413 American adults recruited online through MTurk. Following a similar data screening procedure as in the previous studies, 55 participants were excluded because they did not pay sufficient attention to the manipulation materials or spent significantly more time reading the material than the rest of the sample. Additionally, ten participants spent not even 10 minutes completing the entire survey. One participant raised suspicion about the credibility of the news article, and three participants reported to have close friends or family members from Iran. The 69 participants excluded were approximately evenly distributed across the study's four conditions. 344 participants were retained for subsequent analyses (49% women; age $M = 36$, $SD = 13.35$).

Procedure. This study employed a 2 (target group's role: victim vs. perpetrator) × 2 (ingroup involvement: involved vs. un-involved) experimental design. In conditions where the conflict involved the ingroup, participants read about prisoner abuses committed either by American soldiers against Iranians or by Iranian soldiers against Americans. In conditions where the conflict did not involve the ingroup, participants read about the same prisoner abuses committed either by Australian soldiers against Iranians or by Iranian soldiers against Australians. The news articles were adapted from Study 1 with one minor modification – they provided the names of one perpetrator ("Michael Smith") and his victim ("Amir Mohsen").

Materials.

Perspective-taking with perpetrators. Four items measured the extent to which participants took the perspective of the perpetrator, *Michael Smith* or *Amir Mohsen* (e.g., "*I tried to see things from [Michael Smith's/Amir Mohsen's] point of view;" "It was very easy for me to imagine how [Michael Smith/Amir Mohsen] was feeling."*). The items were worded

slightly differently from those in Studies 1 and 2 because we adapted them to anchor to an identifiable individual perpetrator rather than the perpetrator group.[1]

Support for retributive justice was measured using the same retributive items as in Study 1 for the "U.S.-Iran" conditions, and were adapted to the Australia-Iran context in the conditions where the conflict did not involve participants' ingroup.

Ingroup identification were measured using the same glorification and attachment items as in Study 1.

Results

Joint effects of group role, ingroup involvement, and identification.

Support for retributive justice. We predicted that highly identified Americans would be less supportive of retributive justice when the U.S. rather than Australia was the perpetrator. By contrast, when Iran was the perpetrator, Americans' support for the punishment of Iranian perpetrators is less likely to depend on whether the victim was the U.S. or Australia. To test this hypothesis, we submitted support for retributive justice ($\alpha = .91$, $M = 6.04$, $SD = 1.91$) as a DV to a GLM with target group's role and ingroup's involvement as IVs and identification as a continuous moderating variable.

Our prediction was supported by a significant three-way interaction of identification by target group's role and ingroup involvement, $F(1, 336) = 8.79$, $p = .003$, $\eta^2_p = .026$, *90% CI* [.005, .06] (see Figure 3). Simple effects indicated that high identifiers were significantly less supportive of retributive justice when the *perpetrating* group was the U.S. ($M = 4.52$) rather than Australia ($M = 5.93$), $t(336) = 3.72$, $p < .001$. High identifiers' retributive justice support did not significantly differ, however, depending on whether the *victimized* group was the U.S. ($M = 7.43$) or Australia ($M = 7.35$), $t(336) = -.22$, $p = .825$. Looking at these simple

[1] In this study we also included a parallel set of four items measuring the extent to which participants took the perspective of the victims. Since they are not relevant to the central hypotheses of the current research, the results of victim perspective-taking are not reported here.

effects from a different angle, high identifiers demanded significantly less retributive justice when the U.S. was the perpetrator rather than the victim, $t(336) = 7.57$, $p < .001$, in the U.S.-Iran conflict conditions, replicating the pattern obtained in Studies 1 and 2. The same pattern emerged in the Australia-Iran conflict conditions, but to a significantly lesser degree, $t(336) = 3.97$, $p < .001$. Among low identifiers, neither the manipulation of perpetrator nor the manipulation of victim group significantly affected support for retributive justice, $ts(336) < 1.50$, $ps > .150$.

Besides the predicted three-way interaction, we also obtained a significant two-way interaction between target group's role and identification, $F(1, 336) = 29.05$, $p < .001$, $\eta^2_p = .08$, *90% CI* [.04, .13]. Simple effects revealed that for high identifiers, portraying the target group as the perpetrator ($M = 5.22$) significantly reduced their support for retributive justice, compared to portraying the target group as the victim ($M = 7.39$), $t(336) = -8.25$, $p < .001$. Low identifiers, on the other hand, did not differ depending on the target group's role, $t(336) = -.56$, $p = .578$.

The main effect of group role as the victim or perpetrator on support for retributive justice was also significant, $F(1, 336) = 39.70$, $p < .001$, $\eta^2_p = .11$, *90% CI* [.06, .16]. Portraying the target group as the perpetrator ($M = 5.46$) decreased participants' support for retributive justice, compared to portraying the group as the victim ($M = 6.57$). The interaction between ingroup involvement and identification was also significant, $F(1, 336) = 4.16$, $p = .042$, $\eta^2_p = .01$, *90% CI* [.00, .04]. High identifiers demanded significantly less retributive justice when their ingroup was involved ($M = 5.97$), compared to when it was not ($M = 6.64$), $t(336) = -2.54$, $p = .012$. In contrast, low identifiers did not differ depending on ingroup's involvement in the conflict, $t(336) = .37$, $p = .709$. The main effect of identification was significant as well, $F(1, 336) = 9.25$, $p = .003$, $\eta^2_p = .03$, *90% CI* [.01, .06].

Perspective-taking with perpetrators. We predicted that highly identified Americans would be more likely to take the perspective of perpetrators when the U.S. rather than

Australia committed violence. By contrast, Americans are unlikely to differ in their perspective-taking of Iranian perpetrators depending on whether the victim was the U.S. or Australia. The analysis with perpetrator perspective-taking (α = .75, *M* = 3.68, *SD* = 1.67) as the DV partially supported this hypothesis. Instead of a three-way interaction of identification by group role and ingroup involvement, we obtained a marginally significant two-way interaction between ingroup involvement and group role emerged, $F(1, 336) = 2.95$, $p = .087$, $\eta^2_p = .01$, *90% CI* [.00, .03]. Decomposing this interaction, participants were more likely to take the perspective of perpetrators when the U.S. (*M* = 4.39) rather than Australia (*M* = 3.72) committed violence against Iran, $t(336) = -2.67$, $p = .008$. In contrast, they did not differ significantly in perspective-taking with Iranian perpetrators depending on whether the U.S. (*M* = 3.39) or Australia (*M* = 3.31) was the victim, $t(336) = -.34$, $p = .735$.

There was also a significant two-way interaction between target group's role and identification (Figure 4), $F(1, 336) = 9.38$, $p = .002$, $\eta^2_p = .03$, *90% CI* [.01, .06], again replicating the findings of Studies 1 and 2. Simple effects revealed that among high identifiers, portraying the target group as the perpetrator (*M* = 4.28) significantly increased perspective-taking with the perpetrators, compared to portraying the target group as the victim (*M* = 3.03), $t(336) = 5.04$, $p < .001$. Low identifiers, on the other hand, did not respond differently to perspective-taking depending on group role ($M_{perpetrator} = 3.83$, $M_{victim} = 3.66$), $t(336) = .67$, $p = .501$. This finding suggests that the increased perpetrator perspective-taking among highly identified Americans was not limited to ingroup perpetrators; rather, the effects carried over to perpetrators of a similar outgroup. Most importantly, however, we showed again that high identifiers did engage in more perpetrator perspective-taking when the ingroup committed (rather than suffered) violence.

The analysis also yielded a significant main effect of group role on perspective-taking with perpetrators, $F(1, 336) = 16.72$, $p < .001$, $\eta^2_p = .05$, *90% CI* [.02, .09]; portraying Australia/the U.S. as the perpetrator (*M* = 4.05) increased perspective-taking with the

perpetrators, compared to portraying either country as the victim (M = 3.36). The main effect of ingroup involvement was also significant, $F(1, 336) = 4.74$, $p = .030$, $\eta^2_p = .01$, *90% CI* [.001, .04]; reading about a conflict involving the U.S. (M = 3.87) increased American participants' perspective-taking with the perpetrators, compared to reading about a conflict that did not involve the U.S. (M = 3.51). No other effects reached significance, $Fs(1, 336) < .50$, $ps > .500$, $\eta^2_ps < .002$.

Discussion

This study successfully replicated the main findings of Studies 1 and 2, showing that high identifiers increased their perspective-taking with the perpetrator and reduced their support for retributive justice when the ingroup had committed rather than suffered violence. Additionally, this study further demonstrated that high identifiers increased perpetrator perspective-taking and reduced support for retributive justice in response to ingroup perpetration, as compared to perpetration by a similar outgroup (i.e., Australia) when the ingroup was not involved. In contrast, high identifiers did not respond differently to ingroup- or outgroup-victimization. This finding suggests that high identifiers' differential reactions to ingroup perpetration versus victimization (as shown in Studies 1 and 2) are mainly driven by their past perpetrator rather than victim experiences. While both high and low identifiers took more perspective of the American rather than the Australian perpetrator, only high identifiers' perspective-taking translated into reduced support for retributive justice. In other words, perpetrator perspective-taking only served a harm exonerating function for high, but not low, identifiers.

Additional Figures

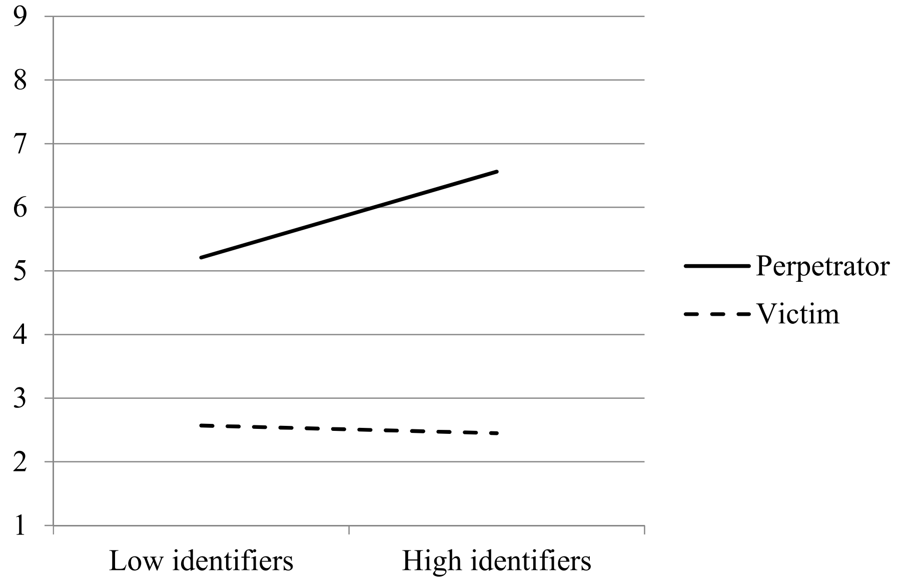

Figure 1. Perspective-taking with perpetrators as a function of ingroup's role as perpetrator or victim and national identification (Study 2).

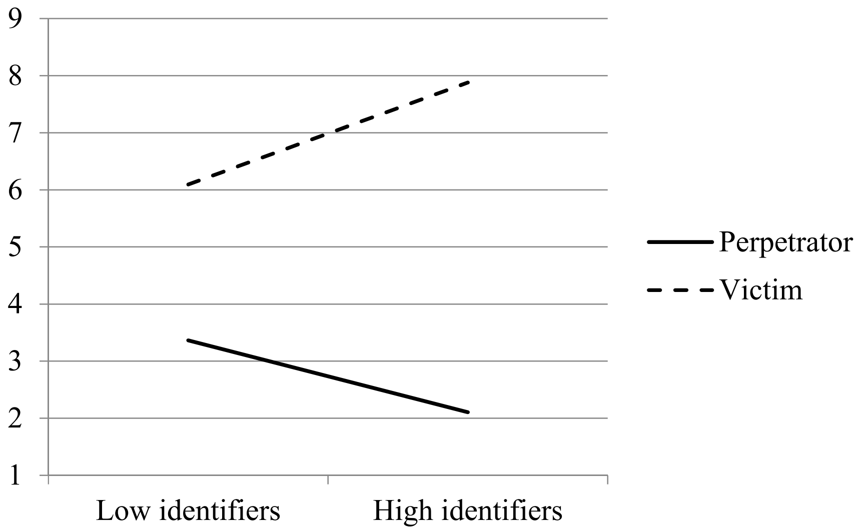

Figure 2. Support for retributive justice as a function of ingroup's role as perpetrator or victim and national identification (Study 2).

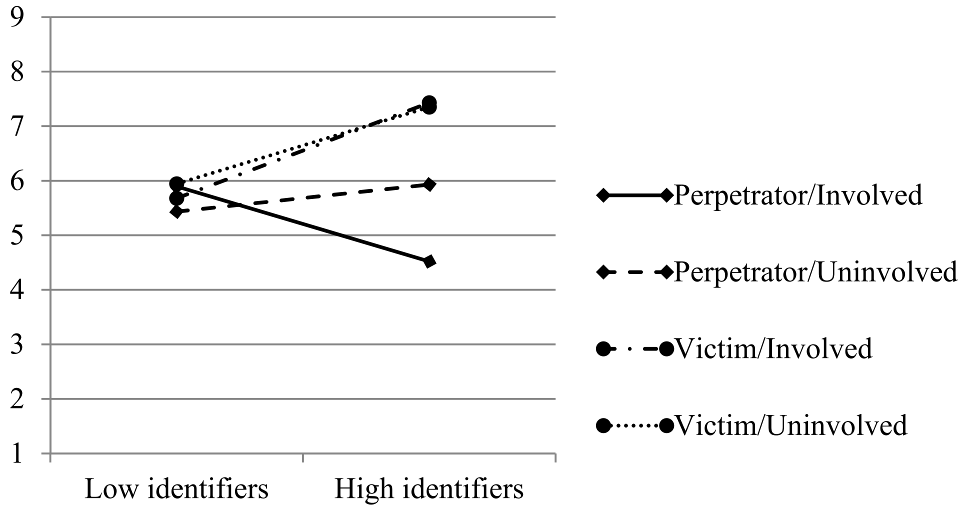

Figure 3. Support for retributive justice as a function of target group's role (perpetrator vs. victim), ingroup's involvement in the conflict (involved vs. uninvolved), and national identification (Additional Study).

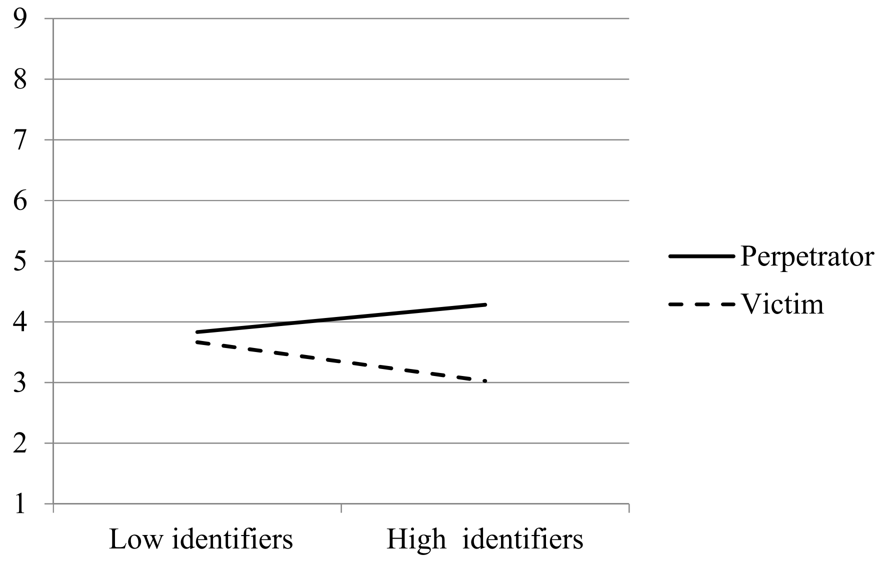

Figure 4. Perspective-taking with perpetrators as a function of target group's role and national identification (Additional Study).
